# The evolving roles of editors and reviewers for nonhuman “authors”: Consequences for the integrity of scientific literature and medical knowledge

**DOI:** 10.34172/jcvtr.025.33733

**Published:** 2025-09-28

**Authors:** Samad Ghaffari, Neda Roshanravan

**Affiliations:** Cardiovascular Research Center, Tabriz University of Medical Sciences, Tabriz, Iran

 Generally, for decades, editors and reviewers have been the core of academic integrity and innovation. Strict standards of quality, originality, and ethical conduct of published research are a primary responsibility of journal editors and reviewers. In recent years, significant advances have been made in the availability of Artificial Intelligence (AI) for various aspects of scholarly publishing.^1^

 The rapidly evolving use of AI tools like large language models (LLMs) for text summarization, ChatGPT for content creation, Midjourney for images, and Pictory for video creation has brought both opportunities and challenges. In other words, generative AI might threaten the principles guiding scholarly publishing but could also be useful in achieving them.^2^ Based on the International Committee of Medical Journal Editors (ICMJE) recommendation, authors, when submitting work to journals, should disclose any use of AI-assisted technologies. Furthermore, they must explain how AI was used, including writing assistance in the acknowledgment section, and data collection, analysis, or figure generation in the methods section. Considering the important fact that AI tools cannot be listed as authors, humans remain responsible for the accuracy, integrity, and originality of the work. With emphasis, authors should carefully review AI-generated content, as it may be incorrect, incomplete, or biased.^3^

 In general, the usage of AI encounters major challenges that extend from manuscript creation into the peer review process. Over the past decade, scientific publications have grown markedly, while there has been a significant shortage of expert reviewers who are willing and able to review manuscripts, particularly in complex domains. One concern in this realm is that reviewers and editors may become excessively dependent on these technological tools. This may reduce the rigor and expert peer reviews needed to evaluate the quality of articles. AI algorithms may also be trained on biased data, which can lead to improper assessment and recommendations.^4,5^

 From a pragmatic standpoint, AI with its ability to use LLMs for summarizing and extracting key points, could make the scientific peer review process faster and more efficient, but testing and standardization are needed to prove its benefits. Given the potential risks, journal editors are proceeding with caution. Based on the ICMJE, “reviewers should be aware that AI can generate authoritative-sounding output that can be incorrect, incomplete, or biased.”^3^ To maintain clarity and trust, the final decision should always be made by humans. Furthermore, the use of these tools in manuscripts makes monitoring and adherence to ethical principles even more important.^6^

## Conclusion

 Although AI tools make manuscript processing and review more efficient, they also necessitate the duties and responsibilities of human editors and reviewers. Establishing clear guidelines is essential for all scientific journals and publishers, requiring authors to disclose their use of AI and obligating editors and reviewers to protect confidential manuscripts from AI tools to maintain integrity and trust.

## Competing Interests

 The authors declare that the research was conducted in the absence of any commercial or financial relationships that could be construed as a potential conflict of interest. The authors declare that they are editorial board members of the Journal of Cardiovascular and Thoracic Research at the time of submission. This had no impact on the peer-review process and the final decision.

## Ethical Approval

 Not applicable.
